# VEXAS, Chediak–Higashi syndrome and Danon disease: myeloid cell endo-lysosomal pathway dysfunction as a common denominator?

**DOI:** 10.1186/s11658-025-00691-0

**Published:** 2025-01-26

**Authors:** Coline Savy, Maxence Bourgoin, Thomas Cluzeau, Arnaud Jacquel, Guillaume Robert, Patrick Auberger

**Affiliations:** 1 University Cote d’Azur, Inserm, C3M, Nice, France; 2 Clinical Hematology Department, Centre Hospitalier Universitaire, Nice, France

**Keywords:** VEXAS, Chediak–Higashi disease, Danon disease, Lysosomes, pH, Neutrophiles, Monocytes, Macrophages, Inflammation

## Abstract

Vacuolization of hematopoietic precursors cells is a common future of several otherwise non-related clinical settings such as VEXAS, Chediak–Higashi syndrome and Danon disease. Although these disorders have a priori nothing to do with one other from a clinical point of view, all share abnormal vacuolization in different cell types including cells of the erythroid/myeloid lineage that is likely the consequence of moderate to drastic dysfunctions in the ubiquitin proteasome system and/or the endo-lysosomal pathway. Indeed, the genes affected in these three diseases UBA1, LYST or LAMP2 are known to be direct or indirect regulators of lysosome trafficking and function and/or of different modes of autophagy. Furthermore, all three genes are highly expressed in the more mature myeloid cells pointing out their likely important function in these cells. LAMP2 deficiency for instance is known to be associated with alterations of lysosome architecture and function. It is thus well established that different cell types from Danon disease patients that harbor invalidating mutations in LAMP2 exhibit giant lysosomes containing undigested materials characteristic of defects in the fusion of lysosomes with autophagosomes, a feature also found in VEXAS and CHS. Other similarities regarding these three diseases include granulocyte and monocyte dysfunctions and a recurrent inflammatory climate. In the present review we discuss the possibility that some common clinical manifestations of these diseases, notably the hematopoietic ones are consecutive to a dysfunction of the endo-lysosomal pathway in myeloid/erythroid progenitors and in mature myeloid cells including neutrophiles, monocytes and macrophages. Finally, we propose reacidification as a way of reinducing lysosome functionalities and autophagy as a potential approach for a better management of these diseases.

## Introduction

### VEXAS syndrome

VEXAS syndrome is an auto-inflammatory disease caused by somatic mutations of the X-linked Ubiquitin like modifier activating enzyme 1 (UBA1) gene primarily affecting initiating Met41 [1]. VEXAS syndrome (VS) is an acronym for Vacuoles; E1 ubiquitin conjugating enzyme; X chromosome; Auto inflammatory and Somatic. The disease occurs in advanced adulthood, around 50 years, preferentially in men and is characterized by both rheumatological and dermatological symptoms [2]. Patients with VS present with autoinflammatory manifestations, including neutrophil-rich lesions reminiscent of Sweet's syndrome and recurrent polychondritis [3, 4]. Loss of UBA1 jeopardizes ubiquitination and triggers immune and inflammatory responses. Inhibition of inflammation using glucocorticoids or anti-IL6 antibodies has shown limited efficacy in VS, while Ruxolitinib and 5-azacytidine (Aza) triggers moderate to sometimes complete responses [5]. Allogeneic hematopoietic stem cell transplantation (HSCT) has shown promising results in treating patients with VS with good overall survival and acceptable incidence of graft versus host reaction and represents to date the only curative option for younger adult suffering VS [6, 7]. Besides rheumatological and dermatological symptoms, hematologic defects are also symptomatic of VS and includes macrocytic anemia, low platelet counts and a predisposition to develop myelodysplastic syndrome (MDS), a pre-leukemic status [8, 9]. The presence of large cytoplasmic vacuoles in myeloid and erythroid precursors in the bone marrow is also a characteristic hallmark of the disease [10, 11]. Both the molecular mechanisms of VS and how UBA1 mutations can lead to vacuolization are currently poorly understood, even though UBA1 deficiency has been previously associated with defects in non-canonic autophagy [12].

### Chediak–Higashi syndrome

Mutations in lysosome trafficking regulator (LYST) are responsible for Chediak–Higashi Syndrome (CHS), a rare hereditary immune deficiency [13]. The main hallmarks of CHS are oculocutaneous albinism, immunodeficiency, bleeding tendency, recurrent infections, multiorgan inflammation, and neurological troubles. Patients with CHS also develop hemophagocytic lymphohistiocytosis (HLH) infiltration of different organs leading to fever, splenomegaly, hepatomegaly and bleeding [14]. HLH is associated with excessive lymphocyte and macrophages responses and is the leading cause of death in CHS patients [15]. CHS is due to nonsense mutations, splice site mutations or even exon deletion of the LYST gene leading to a loss of function [16, 17]. Patients with CHS also develop a characteristic periodontitis [18], a manifestation also found in Papillon-Lefevre syndrome [19] a rare autosomal hereditary disorder due to a deficiency of cathepsin C, a cysteine protease involved in the degradation of proteins imported into the lysosome and leading to defects in chemotaxis and ROS production by neutrophiles. Of note, alterations in CHS patient’s neutrophiles also lead to neutropenia, impaired chemotaxis, delay in phagolysosome fusion and finally defects in bactericidal activity, that results amongst other in periodontal disease [18]. The main diagnostic hallmarks of CHS are i) enlarged lysosomes and lysosome-related organelles in various cell types, including myeloid cells and melanocytes [20, 21] and ii) detection of mutations in the LYST gene. In CHS, autophagosomes seems to be formed in almost normal size and quantities and can fuse to the enlarged lysosomes. However, LYST depletion causes a reduction of the number of small lysosomes per cell with a concomitant increase in large lysosomes, supporting a role for LYST in autophagic lysosome reformation [22]. Thus, the diagnosis of CHS is established by the presence of large cytoplasmic granules in leukocytes and platelets on a peripheral smear and is further confirmed by identification of a pathogenic variant of the LYST gene. HSCT is the treatment of choice for CHS patients [23]. Following HSCT the 5 years overall survival is around 50% [24]. Nevertheless, most of patients suffering CHS die from recurrent infection.

### Danon disease

Danon disease (DD) is a rare X-linked lethal metabolic disorder associated with hypertrophic cardiomyopathy, skeletal weakness and intellectual deficiencies [25]. The disease is more severe in male, where it occurs during childhood and adolescence, than in female who are generally affected in the late adolescence or early adulthood. Currently, there is no treatment for DD patients, excepted heart transplantation with a survival rate at 5 years of 87% on a small cohort of patients [26]. DD is due to genetic mutations in Lysosomal Associated Membrane Protein 2 (LAMP2), an essential component of the lysosome. Liver, muscle, brain and myeloid cells from DD patients exhibit enlarged lysosomes that accumulate undigested cellular materials [27]. These characteristics are reminiscent of defects in degradative pathways including macro-autophagy. Recent data in the literature has led to the conclusion that among the three LAMP2 isoforms (LAMP2A, B and C), LAMP2B deficiency is sufficient and necessary to cause the phenotypes observed in DD cardiomyocytes [28]. Moreover, mice deficient for LAMP2A have no defect in autophagosome-lysosome fusion reinforcing the specific role of LAMP2B and not LAMP2A in DD pathogenesis [28]**.** In conclusion, the three diseases present similar dysfunctions also affecting hematopoietic cells and more particularly cells of the myeloid lineage that have been only partially characterized.

## Loss of protein functions causing VS, CHS and DD

### UBA1

The protein encoded by the UBA1 gene catalyzes the first step in ubiquitin conjugation which is the limiting step for Ubiquitin–Proteasome System (UPS)-dependent degradation of cellular proteins. UBA1 activates ubiquitin by first adenylating its C-terminal glycine residue with ATP, and thereafter linking this residue to the side chain of a cysteine residue in an E1 enzyme, yielding a ubiquitin-E1 thioester and free AMP [29]. The UBA1 gene is located on the X-chromosome in position Xp11.23. Diseases associated with UBA1 deficiency include VS [1] and spinal muscular atrophy, X-linked 2 [30–32]. UBA1 behaves as both a cytoplasmic and nuclear isoform that are initiated at Met41 and Met67, respectively. UBA1 is a 110 to 120 kDa protein depending on the isoform and consists of five functional domains including an N-terminal inactive adenylation domain (IAD), the first catalytic cysteine half domain, the active adenylation domain (AAD), the second catalytic cysteine half domain, and C-terminal ubiquitin fold domain. The IAD and AAD domain adenylate the first ubiquitin, and transfer ubiquitin to the E1-like enzyme. Mutations in UBA1 lead to loss of function proteins with altered catalytic activity.

### LYST

The Lysosomal Trafficking Regulator LYST gene located on chromosome 1 encodes a protein that regulates intracellular protein trafficking in endosomes and fusion of intracellular vesicles such as lysosomes [33, 34]. LYST exerts important functions in immune cells. For instance, in cytotoxic T-cells and natural killer cells, LYST controls the size, number and exocytosis of lytic granules [35]. In macrophages and dendritic cells, it regulates phagosome maturation by controlling the conversion of early phagosomal compartments into late phagosomes [36]. LYST also participates in TLR3- and TLR4-induced production of pro-inflammatory cytokines by regulating the endosomal TLR3-TICAM1/TRIF and TLR4-TICAM1/TRIF signaling pathways [37]. Because of its huge size of around 430 kDa, LYST protein function has remained enigmatic for a long time, but pieces of evidence indicate a role in the lysosomal endosomal pathway and more precisely in autophagic lysosome reformation [22]. Interestingly, two animal models with acquired or spontaneous mutations of the LYST gene have been reported, the *beige* mutation that occurs spontaneously in a strain of C57BL/6J mice phenocopies the features of the human disease [38] and established mutants of the *mauve* gene that codes for drosophila LYST that recapitulate the cellular defects associated with CHS [39, 40]. As the main hallmark of CHS is immunological dysfunction, it is noteworthy that lysosomes in bone marrow-derived macrophages and melanosomes of the skin epidermal melanocytes and eye-pigmented cells from beige mice showed abnormal tubular morphology and trafficking [41].

### LAMP2

The LAMP2 gene is located on the X chromosome in position Xq24 [42]. It encodes a highly glycosylated 120 kDa glycoprotein of the lysosome accounting with LAMP1 for 50% of all lysosomal membrane proteins [43–45]. LAMP2 behaves as three different isoforms generated by alternative splicing of exon 9, namely LAMP2A, LAMP2B and LAMP2C [46]. Each isoform exerts a unique function in the regulation of different modes of autophagy. LAMP2A acts as the receptor for chaperone-mediated autophagy, a highly selective form of autophagy in which proteins endowed with a KFERQ motif are directed to the lysosome for degradation [47]. LAMP2B has a unique role in the fusion of autophagosome with lysosome in the process of macro autophagy [45]. Finally, LAMP2C is thought to participate in the lysosomal degradation of DNA and RNA through a process known as DNA/RNAphagy [48]. LAMP2 deficiency is associated with a X-linked disorder called DD whose clinical manifestations includes hypertrophic cardiomyopathy, myopathy and intellectual disability [27]. This disease is more sever in male than in female and most male patients die around 25 years old. The role of LAMP2 deficiency as a driver of DD is exemplified by LAMP2-deficient mice models that recapitulates accurately the main characteristics of the disease [49, 50]. However, the specific role of each of the three LAMP2 isoforms in DD is not fully known. Most of the genetic studies on the role of LAMP2 have been performed with the LAMP2A isoform, which is involved in a great number of pathologies including atherosclerosis, aging, liver diseases and neurodegenerative disorders [51–54]. The absence of mouse models with specific deletion of LAMP2B had precluded any analysis of its potential role in DD. However, the discovery that some DD patients carry mutations that only affect LAMP2B function is a strong argument for an indispensable role of this isoform in the disease [55]. In addition, the characteristic transmission electronic microscopy images of lysosomes from LAMP2 deficient mice showing giant lysosomes harboring undigested material strongly argues for a role of LAMP2B and not LAMP2A and C in DD.

## UBA1, LYST and LAMP2 expression in hematopoietic stem cells (HSCS) and the myeloid lineage

**Fig. 1:**
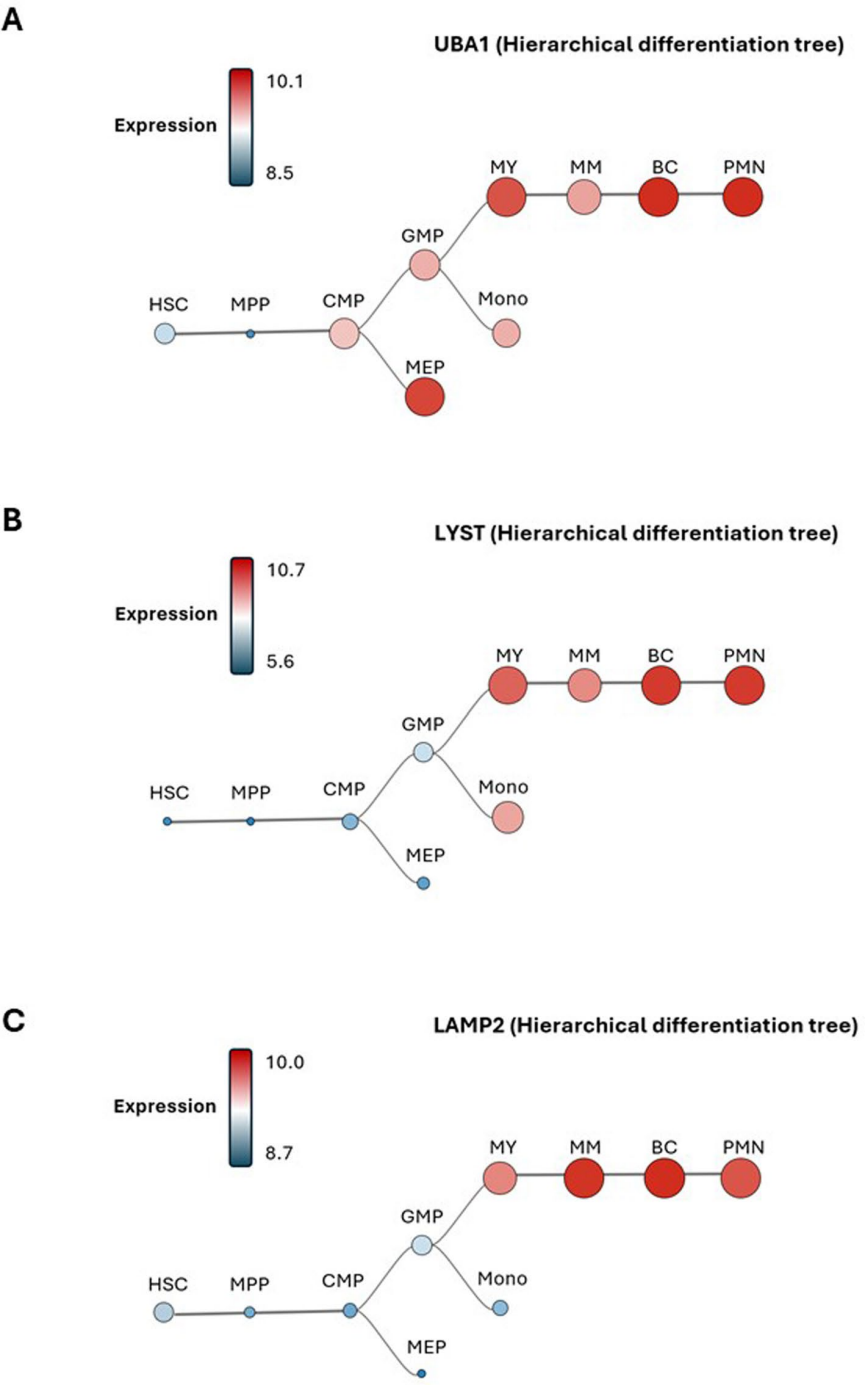
Expression of UBA1, LYST and LAMP in the myeloid lineage. **A** UBA1, LYST and LAMP2 mRNA expression follow identical trajectories during development of the myeloid lineage. Relatively low in hematopoietic stem cells (HSC) and multipotent myeloid progenitors (MMP), granulocytic/monocytic progenitors (GMP), monocytes (Mono) and drastically decreases in polymorphonuclear (MPP), UBA1, LYST and LAMP2 mRNA expression increases in polymorphonuclear neutrophiles (PMN). The data used to generate these Hierarchical Differentiation Tree was obtained from a cohort known as “normal hematopoiesis with AMLs” through the bloodspot website (https://bloodspot.eu/)

Using Bloodspot, we analyzed the level of expression of UBA1, LYST and LAMP2 mRNA in the myeloid cell differentiation tree from HSC to mature myeloid cells. UBA1 mRNA expression is relatively low in HSCs and in myeloid progenitors. UBA1 mRNA expression then increases at the common multipotent (CMP) and granulocyte/monocytes (GMP) progenitor stages. Myelocytes (MY), myeloid/erythroid progenitors (MEP), metamyelocytes (MM), band cells (BC) and polymorphonuclear neutrophiles (PMN) express high to very high level of UBA1 mRNA (Fig. 1A), while an intermediate expression is detected in monocytes. Of note, this high pattern of expression in PMN is compatible with the defects observed in granulocytes from CHS patients. RNA seq analysis of myeloid differentiation tree shows very low expression of LYST mRNA in HSCs and precursors (Fig. 1B). LYST mRNA expression then increases during terminal differentiation of the myeloid lineage and more particularly in MY, BC and PMN, while an intermediate expression is also detected in MM and monocytes (Fig. 1B). This is also consistent with the defect observed in neutrophiles and monocytes in this disease. LAMP2 mRNA expression is relatively high in HSCs, in agreement with its important role in HSC homeostasis. LAMP2 mRNA expression is also very high in MM, BC and PMN (Fig. 1C). Accordingly, we have recently reported that in human, neutrophiles express the highest levels of LAMP2 protein expression among all blood cells [56]. Interestingly, LAMP2 mRNA expression phenocopies LYST and UBA1 mRNA expression in the myeloid differentiation tree (Fig. 1C). To conclude the pattern of expression of UBA1, LYST and LAMP2 is very similar in the myeloid lineage, more particularly in the more mature cells, which may explain some of the overlapping hematopoietic manifestations found in neutrophiles and monocytes of VS, CHS and DD patients.

## Similarities in haematological disease manifestations

**Fig. 2:**
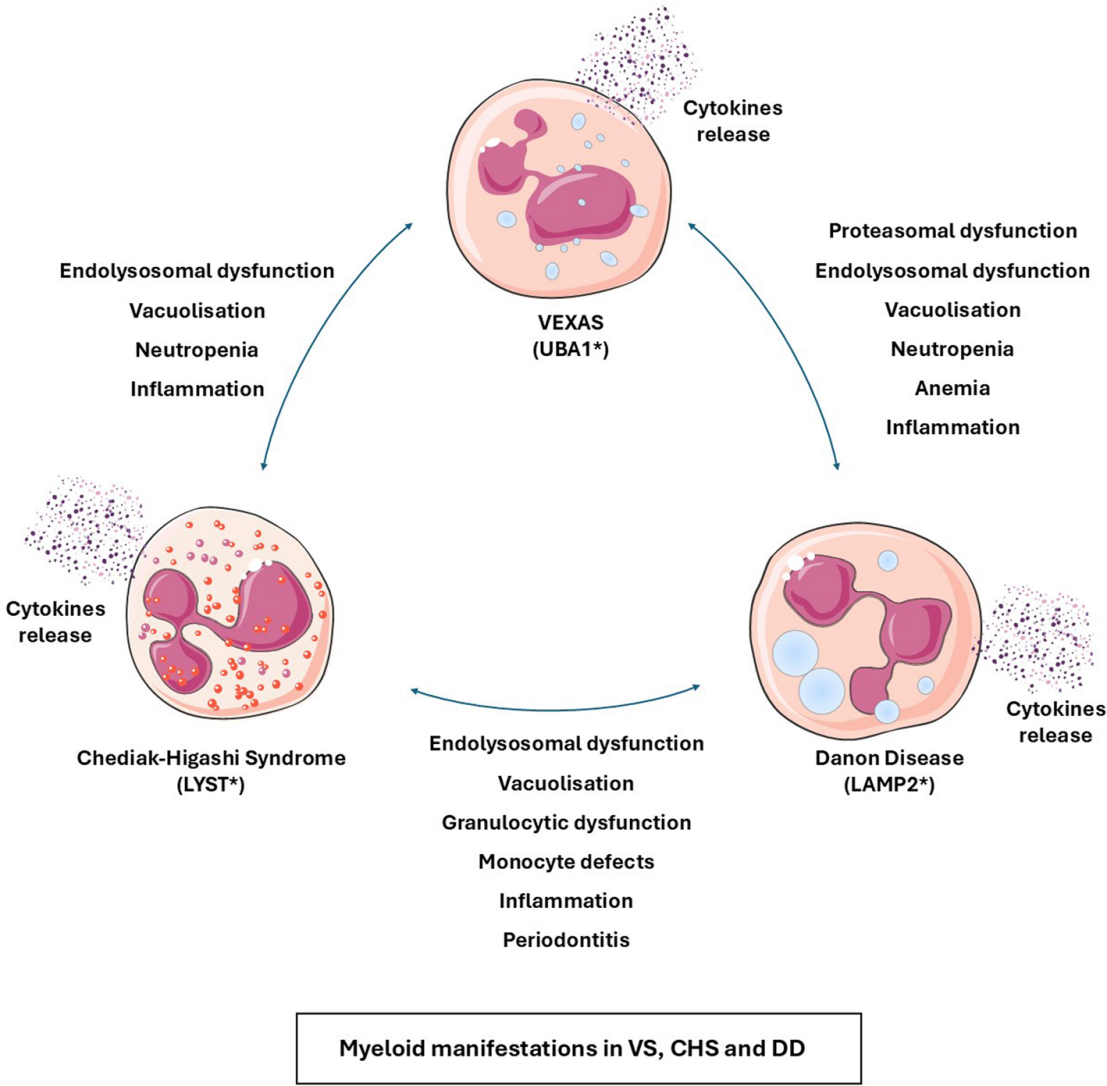
Similarities in hematopoietic manifestations in VS, CHS and DD. VS, CHS and DD are characterized by inactivating mutations in UBA1, LYST and LAMP2, respectively. The main similarities between these three clinical conditions are a recurrent dysfunction of the proteosomal and/or the endolysosomal pathways which leads to an accumulation of small to giant vacuoles, corresponding to non-functional lysosomes. Granulocytic dysfunction and neutropenia are also common features of VS, CHS and DD, while anemia is also consistently observed in VS. Finally, diverse inflammatory manifestations and increase production of inflammatory cytokines are also hallmarks of all three diseases

Although highly different in nature and clinical manifestations, all three diseases are characterized morphologically by the accumulation of enlarged vesicles containing undigested cellular materials in the cytoplasm of many cell types. This is particularly true regarding cells issued from the myeloid lineage whose dysfunction may be responsible for the impairment of granulocyte functions and pro-inflammatory conditions characteristic of these diseases. Although these defects have not been characterized in detail, they likely correspond to moderate to severe dysfunction of the endo-lysosomal pathway in myeloid cells leading to impaired mechanisms of autophagy and defects in granulocyte functionality. The hematological manifestations that occur in all three diseases could be thus the direct consequence of the deregulation of lysosomal degradation and/or trafficking. Morphologically, erythroid and myeloid cells in VS, CHS and DDS exhibit large or even giant lysosomes containing undigested materials that are also found in cells treated with lysosomotropic drugs including chloroquine, hydroxychloroquine or V-ATPase inhibitors such as bafilomycin A1 and concanamycin A, that all increase intra lysosomal pH and inhibit fusion of autophagosomes with lysosomes. Thus, one hypothesis to explain these common features could be a defect in the regulation of intra-lysosomal pH that warrants to be analyzed in detail in all three diseases. Interestingly, as it is the case for CHS, impaired phagosomal maturation also leads to a periodontal disease in LAMP2 deficient mice [57] comforting another similarity between these two diseases. Exacerbated inflammatory responses are also a hallmark of VS, CHS, DD and LAMP2-deficient mice. RNAseq analysis identified some similarities in cytokine expression in the blood of VS patients [9] and the bone marrow and blood of LAMP2 deficient mice [58]. In conclusion, Fig. 2 recapitulates the main similarities in myeloid manifestations of VS, CHS and DD.

## Abnormal endolysosomal function as a common feature?

While very different in their clinical presentation and regarding the types of organs affected, VS, CHS and DD all share defects in the development and function of the erythroid/myeloid lineage. As previously mentioned, these defects are likely due to alterations in the endo-lysosomal compartment leading to impaired catabolism related to different processes including macro autophagy, chaperone-mediated autophagy and the ubiquitin proteasome system. Accordingly, VS is characterized by a mutation of UBA1, a main E1-ligase involved in protein ubiquitination, CHS by mutations in LYST a protein controlling the size and numbers of lysosomes and DD by mutations in LAMP2, a key protein involved in lysosome integrity and function and in the regulation of different forms of autophagy. These defects are particularly prevalent in myeloid cells such as monocytes, macrophages and neutrophiles that expressed high level of UBA1, LYST and LAMP2. In normal condition, lysosomes are acidic organelles with an intracellular pH of 4.5–5. This low pH ensures an optimal function of the hydrolytic enzymes responsible for catabolism and is also required for the proper function of different modes of autophagy. The question that arises from these observations is whether increased intra-lysosomal pH is responsible for the defects observed in hematopoietic cells in these three diseases, and if so, is it possible to restore the functionality of blood cells, more particularly monocytes and neutrophiles using drugs capable of reacidifying intra-lysosomal pH?

## Reacidification of lysosomes as a therapeutic strategy in VEXAS, CHS and DD?

Lysosomes play an essential role in different cellular processes including endocytosis, phagocytosis, nutrient sensing, energy metabolism and autophagy [59, 60]. Lysosomal catabolic functions are ensured by a panel of hydrolytic enzymes that degrade engulfed macromolecules and whose activity is optimal at acidic pH. Defects of lysosomal acidification are involved in the pathogenesis of different illnesses such as neurodegeneration, cancer, infectious diseases and metabolic disorders [61]. Lysosomes and lysosome-related organelles are acidified by a family of proton pumps, referred as the vacuolar H (+)-ATPases (v-ATPase) [62–64]. The electrogenic v-ATPase creates electrical and chemical gradients across lysosomal membranes. As mentioned previously, lysosomes from DD patients and LAMP2 knock-out mice exhibit an abnormally basic pH responsible for profound defects in cellular catabolism and autophagy. Concerning the pathophysiology of VS and CHS, although lysosome dysfunctions are suspected to play a role, it is not known whether lysosome acidification defects are involved. Of note, electron microscopy images of lysosomes in VS and CHS are reminiscent of those found in numerous cell types from DD patients and LAMP2-deficient mice, suggesting a dysfunction due to abnormal acidification. Nevertheless, modulating lysosomal pH appears as an attractive and promising therapeutic strategy to restore at least partially lysosomal functions in VS, CHS and DD. Of note, while a plethora of agents can increase lysosomal pH including Chloroquine, Hydroxychloroquine, Bafilomycine A1, Concanamycine A, some chemotherapeutic drugs (Doxorubicine) and targeted therapies due to their high pKa values, only a small number of compounds have been described so far to be able to reduce it. Among them are PLGA and PLA nanoparticles, 4-aminopyridine a general inhibitor of potassium channels, prosapogenin A and mTORC1 inhibitors.

### Reacidification of lysosomes using PLGA and PLA nanoparticles

Lysosomal dysfunctions including impairment of lysosomal acidity have been associated with neurodegenerative diseases and Non-Alcoholic Fatty Liver Disease (NAFLD) [65, 66]. Based on the notion that neutrophile and monocyte dysfunctions in VS, CHS and DD are associated with altered lysosomal pH, strategies to restore normal lysosomal acidity could represent a therapeutic opportunity in all three diseases. In this line, acidic nanoparticles such as poly-lactide-co-glycolic acid (PLGA) and polylactic acid (PLA) have emerged as a new option to target specific tissues and cell types and serve as drug delivery vehicles. Nanoparticles are mostly engulfed by monocytes/macrophages leading to degradation of their cargo in lysosomes. As myeloid cell lysosomes from VS, CHS and DD are thought to be defective likely through increased intra-lysosomal pH, reacidification of lysosomes could thus appeared as a pertinent therapeutic strategy. In agreement with this possibility, PLGA and PLA have shown promising results in atherosclerosis and NAFLD. Indeed, Zhang et al. administered two commonly used acidic nanoparticles, PLGA and PLA both in vitro and in vivo. They established that PLGA-based nanoparticles triggered reacidification of lysosomes leading to enhanced lysosomal degradation, promotion of autophagy and reduced apoptosis and inflammasome activation [67]. In vivo, PLGA also accumulate in lysosomes and trigger lysosomal acidification of macrophages in atherosclerotic plaques improving atherosclerotic lesions [67]. In the same line, Zeng et al., reported that restoration of lysosomal acidification rescues autophagy and metabolic dysfunction in NAFLD [66].

### Targeting TMEM175 using 4-aminopyridine

TMEM175 has been initially identified as a constitutively active leak-like potassium channel that regulates lysosomal pH stability and fusion of lysosomes and autophagosomes during autophagy, since these functions are dysregulated in the lysosomes of TMEM175 KO mice [68]. TMEM175 deficiency results in an unstable lysosomal pH, which leads to decreased lysosomal catalytic activity and impaired autophagosome clearance [69]. More recently, it was established that TMEM175 is a proton-activated proton channel able to further acidify lysosomal lumen [70]. A missense single nucleotide polymorphism (Met393Thr) in TMEM175 also affects lysosomal pH and autophagosome clearance [71]. In summary, recent findings reveal the role of TMEM175 as a proton-activated proton channel of the lysosomal membrane. Of note, it has been recently reported that lysosomal LAMP1 and 2 proteins are crucial for the regulation of lysosomal pH through direct inhibition of the TMEM175 channel [72]. Indeed, LAMP1 or LAMP2 binding to TMEM175 leads to inhibition of proton efflux contributing to intra-lysosomal acidification, which is conducive to a better activation of lysosomal hydrolases. 4-aminopyridine (Fampridine) which is a general inhibitor of voltage-dependent potassium channels used to limit spastic gait disorders in patients with multiple sclerosis could be therefore tested to restore the intra-lysosomal pH in these diseases.

### Prosapogenin A

Prosapogenin A (PA), a steroid saponin and bioactive compound has shown potential as an antineoplastic agent against various human tumors [73]. It has been recently reported that PA promotes lysosomal membrane permeabilization (LMP), leading to the release of cathepsins that activate caspase 8/3 to cleave Gasdermin E. PA also upregulates three key functional subunits of V-ATPase-ATP6V1A, ATP6V1B2, and ATP6V0C, resulting in lysosomal over-acidification. This over-acidification exacerbates LMP and subsequent lysosomal damage. Neutralization of lysosomal lumen acidification or inhibition/knockdown of these V-ATPase subunits attenuates PA-induced lysosomal damage, pyroptosis, and growth inhibition of thyroid cancer cells, highlighting the critical role for lysosomal acidification and LMP in PA's anticancer effects. PA may thus act as a V-ATPase agonist targeting lysosomal acidification, and as such could represent a new potential therapeutic option in diseases associated with defects in lysosomal acidification.

### Azacitidine

Azacitidine (Aza), an hypomethylating agent, is the leading treatment for patients suffering MDS and AML and has been recently used to treat VS patients. Several cases of molecular responses to Aza have been reported in VS patients and among them two patients were shown to have complete clinical response [74–77]. Interestingly, in human-induced pluripotent stem cell-derived cardiomyocytes established from a female patient with DD, administration of azacitidine was shown to reactivate the silent LAMP2 allele, to improve deficient autophagy and to ameliorate contractile cardiac activity in cardiomyocytes [78]. We have previously reported that azacytidine can increase LAMP2 expression in MDS and AML patient’s bone marrow cells [56]. As AZA has shown some efficacy in VS patients it would be interesting in further studies to assess whether it may induce lysosomal acidification of hematopoietic cells in patients suffering VHS and DD.

### Targeting mTORC1

Inhibitors of mTORC1 including Torin1 and AZD8055 has been reported to trigger partial reacidification of lysosome [79]. Indeed, both drugs trigger a significant decrease of intra-lysosomal pH in different cell lines *and *in vivo*.* According to our hypothesis that reacidification of lysosomes could be a therapeutic window in VS, CHS and DD, clinically available mTORC1 inhibitors could be tested in these diseases.

## Conclusions and future perspectives

Exploring the molecular mechanisms underlying the dysfunction of the lysosomal/endosomal pathway in the myeloid lineage from VS, CHS and DD will certainly help to better understand their pathophysiology and to propose new therapeutic opportunities. These dysfunctions stand out to macro-autophagy, chaperone-mediated autophagy, fusion/fission of lysosomes with endosomes, autophagic lysosomal reformation and more globally in endosomal/lysosomal trafficking. We hypothesize that similarities in some myeloid and erythroid manifestations of these diseases such as anemia, neutropenia, and monocyte/macrophage dysfunctions could be explained (i) by their high expression and functional importance in the erythroid/myeloid lineage, (ii) by mutations affecting directly or indirectly the proteasomal and endo-lysosomal pathways and by (iii) a general deregulation of the lysosomal pH consecutive to these different mechanisms. We propose that abnormal stability of the lysosomal pH could be one of the main causes of lysosomal dysfunction in VS, CHS and DD. It was noted previously that a substantial fraction of lysosomes in mammalian cells, although containing degradative enzymes, has a more elevated pH and are catabolically less active [80]. In this context, reacidification of lysosomes could represent a promising therapeutic option for VS, VHS and DD. Finally, it has been reported that lysosomal pH is regulated in a sex-dependent manner in cells of the myeloid lineage [81], a finding of interest, notably regarding the fact that both VS and DD are X-linked diseases and that incidence of both diseases is higher in male than in female. A systematic analysis of intra-lysosomal pH of immune cells in both male and female patients, more particularly myeloid cells thus warrants further examination to better understand the importance of lysosomal alterations involved in the pathophysiology of these three diseases.

## Data Availability

Not applicable.

## Abbreviations

CHS: Chediak Higashi syndrome
DD: Danon disease
VS: Vexas syndrome
MDS: MeyloDysplastic syndrome
NAFLD: Non alcoholic fatty liver disease
UBA1: Ubiquitin Like Modifyer Activating Enzyme 1
LYST: Lysosomal trafficking regulator
LAMP1: Lysosomal Associated Membrane protein 2
LAMP2: Lysosomal Associated Membrane Protein 2
HSC: Hematopoietic stem cell
Vav1: Vav guanine nucleotide exchange factor 1
CMP: Common myeloid progenitor
GMP: Granulocyte-monocyte progenitor
MEP: Megakaryocytes-erythroid progenitor
MY: Myelocyte
MM: Metamyelocyte
BC: Band cell
PMN: PolyMorphoNuclear
TGCA: TGCA AML cohort
CMA: Chaperone-mediated autophagy
LAMP2: Lysosomal-Associated Membrane Protein 2
mTORC1: Mammalian Target Of Rapamycin Complex 1
TMEM175: Transmembrane protein 175
PLGA: Poly-lactide-*co*-glycolic acid
PLA: Polylactic acid
PA: Prosapogenine A
LMP: Lysosomal membrane permeabilization
IAD: Inactive adenylation domain
AAD: Active adenylation domain
